# Comparative prognostic importance of measures of left atrial structure and function in non-ischaemic dilated cardiomyopathy

**DOI:** 10.1093/ehjci/jeae080

**Published:** 2024-03-16

**Authors:** Daniel J Hammersley, Srinjay Mukhopadhyay, Xiuyu Chen, Leanne Cheng, Richard E Jones, Lukas Mach, Lara Curran, Momina Yazdani, Alma Iacob, Amrit S Lota, Zohya Khalique, Antonio De Marvao, Resham Baruah, Kaushik Guha, James S Ware, John Gregson, Shihua Zhao, Dudley J Pennell, Upasana Tayal, Sanjay K Prasad, Brian P Halliday

**Affiliations:** National Heart and Lung Institute, Imperial College London, Sydney Street, London SW3 6NP, UK; Royal Brompton and Harefield Hospital, Guy’s & St Thomas’ NHS Foundation Trust, London, UK; National Heart and Lung Institute, Imperial College London, Sydney Street, London SW3 6NP, UK; Royal Brompton and Harefield Hospital, Guy’s & St Thomas’ NHS Foundation Trust, London, UK; State Key Laboratory of Cardiovascular Disease, National Center for Cardiovascular Diseases, Chinese Academy of Medical Sciences, Peking Union Medical College, Fuwai Hospital, Beijing, China; National Heart and Lung Institute, Imperial College London, Sydney Street, London SW3 6NP, UK; Royal Brompton and Harefield Hospital, Guy’s & St Thomas’ NHS Foundation Trust, London, UK; National Heart and Lung Institute, Imperial College London, Sydney Street, London SW3 6NP, UK; Royal Brompton and Harefield Hospital, Guy’s & St Thomas’ NHS Foundation Trust, London, UK; Essex Cardiothoracic Centre, Basildon, UK; Anglia Ruskin University, Chelmsford, UK; National Heart and Lung Institute, Imperial College London, Sydney Street, London SW3 6NP, UK; Royal Brompton and Harefield Hospital, Guy’s & St Thomas’ NHS Foundation Trust, London, UK; National Heart and Lung Institute, Imperial College London, Sydney Street, London SW3 6NP, UK; Royal Brompton and Harefield Hospital, Guy’s & St Thomas’ NHS Foundation Trust, London, UK; National Heart and Lung Institute, Imperial College London, Sydney Street, London SW3 6NP, UK; Royal Brompton and Harefield Hospital, Guy’s & St Thomas’ NHS Foundation Trust, London, UK; National Heart and Lung Institute, Imperial College London, Sydney Street, London SW3 6NP, UK; Royal Brompton and Harefield Hospital, Guy’s & St Thomas’ NHS Foundation Trust, London, UK; National Heart and Lung Institute, Imperial College London, Sydney Street, London SW3 6NP, UK; Royal Brompton and Harefield Hospital, Guy’s & St Thomas’ NHS Foundation Trust, London, UK; National Heart and Lung Institute, Imperial College London, Sydney Street, London SW3 6NP, UK; Royal Brompton and Harefield Hospital, Guy’s & St Thomas’ NHS Foundation Trust, London, UK; British Heart Foundation Centre of Research Excellence, School of Cardiovascular and Metabolic Medicine and Sciences, King's College London, London, UK; Department of Women and Children's Health, King’s College London, London, UK; Medical Research Council Laboratory of Medical Sciences, Imperial College London, UK; Royal Brompton and Harefield Hospital, Guy’s & St Thomas’ NHS Foundation Trust, London, UK; Portsmouth Hospitals NHS Trust, Portsmouth, UK; National Heart and Lung Institute, Imperial College London, Sydney Street, London SW3 6NP, UK; Royal Brompton and Harefield Hospital, Guy’s & St Thomas’ NHS Foundation Trust, London, UK; Medical Research Council Laboratory of Medical Sciences, Imperial College London, UK; London School of Hygiene and Tropical Medicine, London, UK; State Key Laboratory of Cardiovascular Disease, National Center for Cardiovascular Diseases, Chinese Academy of Medical Sciences, Peking Union Medical College, Fuwai Hospital, Beijing, China; National Heart and Lung Institute, Imperial College London, Sydney Street, London SW3 6NP, UK; Royal Brompton and Harefield Hospital, Guy’s & St Thomas’ NHS Foundation Trust, London, UK; National Heart and Lung Institute, Imperial College London, Sydney Street, London SW3 6NP, UK; Royal Brompton and Harefield Hospital, Guy’s & St Thomas’ NHS Foundation Trust, London, UK; National Heart and Lung Institute, Imperial College London, Sydney Street, London SW3 6NP, UK; Royal Brompton and Harefield Hospital, Guy’s & St Thomas’ NHS Foundation Trust, London, UK; National Heart and Lung Institute, Imperial College London, Sydney Street, London SW3 6NP, UK; Royal Brompton and Harefield Hospital, Guy’s & St Thomas’ NHS Foundation Trust, London, UK

**Keywords:** dilated cardiomyopathy, left atrium, strain, risk prediction

## Abstract

**Aims:**

This study aimed to compare the association between measures of left atrial (LA) structure and function, derived from cardiovascular magnetic resonance (CMR), with cardiovascular death or non-fatal heart failure events in patients with non-ischaemic dilated cardiomyopathy (DCM).

**Methods and results:**

CMR studies of 580 prospectively recruited patients with DCM in sinus rhythm [median age 54 (interquartile range 44–64) years, 61% men, median left ventricular ejection fraction 42% (30–51%)] were analysed for measures of LA structure [LA maximum volume index (LAVI_max_) and LA minimum volume index (LAVI_min_)] and function (LA emptying fraction, LA reservoir strain, LA conduit strain (LACS), and LA booster strain]. Over a median follow-up of 7.4 years, 103 patients (18%) met the primary endpoint. Apart from LACS, each measure of LA structure and function was associated with the primary endpoint after adjusting for other important prognostic variables. The addition of each LA metric to a baseline model containing the same important prognostic covariates improved model discrimination, with LAVI_min_ providing the greatest improvement [*C*-statistic improvement: 0.702–0.738; *χ*^2^ test comparing likelihood ratio *P* < 0.0001; categorical net reclassification index: 0.210 (95% CI 0.023–0.392)]. Patients in the highest tercile of LAVI_min_ had similar event rates to those with persistent atrial fibrillation. Measures of LA strain did not enhance model discrimination above LA volumetric measures.

**Conclusion:**

Measures of LA structure and function offer important prognostic information in patients with DCM and enhance the prediction of adverse outcomes. LA strain was not incremental to volumetric analysis for risk prediction.

## Graphical Abstract

**Graphical Abstract:**
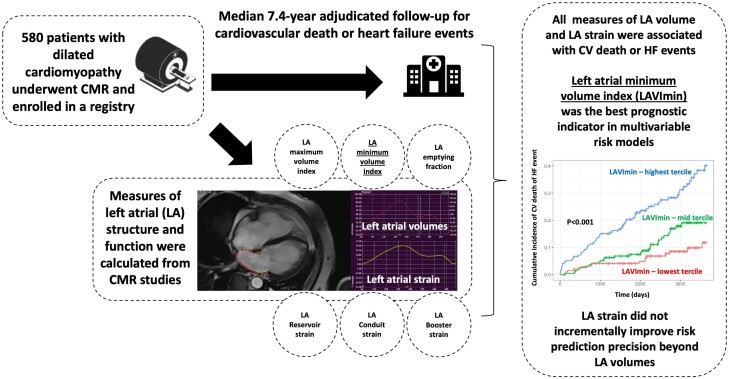

**See the editorial comment for this article ‘Left atrial volume as risk marker: is minimum volume superior to maximum volume?’, by O.A. Smiseth *et al.*, https://doi.org/10.1093/ehjci/jeae136.**

## Introduction

Non-ischaemic dilated cardiomyopathy (DCM) is a major contributor to the global burden of heart failure (HF). Whilst advances in treatment have underpinned improved clinical outcomes,^1^ DCM remains the leading indication for cardiac transplantation globally.^2^ A major challenge within this group lies in risk prediction, where greater precision may guide more personalized therapy. Left atrial (LA) maximum volume index (LAVI_max_) is a component of imaging protocols across different modalities and is associated with adverse HF outcomes in DCM.^3,4^ However, it remains unclear whether LAVI_max_ is superior to alternative measures of LA structure and function, which are increasingly available due to improved access and growing expertise in advanced imaging. Such novel measures include LA minimum volume index (LAVI_min_), LA emptying fraction (LAEF), and phasic LA strain.^5^ The prognostic importance of alternative LA metrics has been evaluated in other conditions; LAVI_min_ was a more powerful predictor of cardiovascular (CV) outcomes than LAVI_max_ in cohorts of patients with HF with preserved ejection fraction and hypertrophic cardiomyopathy (HCM).^6,7^ LAEF predicted survival in a large cohort of patients with HF of mixed aetiology.^8^ LA strain quantifies mechanical atrial deformation aligned to different phases of the cardiac cycle, including LA reservoir strain (LARS) during passive atrial filling, LA conduit strain (LACS) during passive atrial emptying, and LA booster strain (LABS) during active atrial contraction.^9^ LACS was independently associated with adverse CV outcomes in DCM in a further study.^10^ However, clarification is required regarding the additive prognostic value of each LA parameter in this population. We studied the incremental predictive value of measures LA structure and function from cardiovascular magnetic resonance (CMR) in relation to a composite endpoint of CV death or non-fatal HF events in a large cohort of patients with DCM.

## Methods

### Study population

Consecutive patients referred for a CMR between 2009 and 2016 from our clinical service and a network of surrounding hospitals were prospectively enrolled into the Royal Brompton Hospital Cardiovascular Research Centre (RBH CRC) Biobank. The study complied with the Declaration of Helsinki and was approved by the National Research Ethics Service (South Central Hampshire B Research Ethics Committee, Reference 19/SC/0257). All participants provided written consent. Inclusion criteria were confirmed DCM, defined as reduced left ventricular ejection fraction (LVEF) and increased indexed LV end-diastolic volume in relation to age- and sex-adjusted nomograms.^11^ Exclusion criteria were significant ischaemic heart disease (IHD) (defined as stenosis > 50% in a major epicardial coronary artery, inducible ischaemia on functional testing, or prior coronary revascularization), adverse loading conditions (uncontrolled hypertension or severe primary valve disease), congenital heart disease, active myocarditis, or an alternative cardiomyopathy. Patients with atrial fibrillation (AF) at the time of CMR (*n* = 105) were excluded from the primary analysis, as a reliable data set for LA strain could not be obtained in such patients. These patients were later included as a comparator group in the survival analysis.

### Cardiovascular magnetic resonance

All patients underwent a CMR scan at 1.5 Tesla (Sonata/Avanto, Siemens, Erlangen, Germany). Breath-hold steady-state free precession sequences were performed to produce long- and short-axis cine images. Gadopentetate dimeglumine or gadobutrol (0.1 mmol/kg) was injected intravenously and an inversion recovery gradient echo sequence was undertaken to acquire the LGE images at 10 min. Left and right ventricular volumes and LV mass were measured using CMRtools (Cardiovascular Imaging Solutions, London, UK) and indexed to body surface area (BSA). Cine images were analysed for LA structure and function using Medis Qstrain (v2.0) and QMass (v8.1) on Medis Suite v3.1 (Medis Medical Imaging Systems, Leiden, the Netherlands) by a single expert operator blinded to clinical outcomes. This involved delineation of the mitral annulus and LA roof in two- and four-chamber views at LV end-diastole and end-systole, from which the LA endocardial borders were semi-automatically contoured and manually adjusted. LAVI_max_ and LAVI_min_ were derived using the biplane area-length method, indexing absolute atrial volumes to BSA. LA appendage and pulmonary veins were excluded. LAEF was derived as ([LAV_max_ − LAV_min_]/LAV_max_) × 100. LA contours were tracked automatically via the Qstrain package and phasic strain parameters were obtained for LARS, LACS, and LABS from strain curves. Only a single baseline CMR scan was analysed per patient; follow-up CMR scans in the small subset for whom this was available were not analysed. Reproducibility was assessed in 30 randomly selected cases, which were repeated by the primary operator for intra-observer variation and by a second independent expert operator for inter-observer variation.

### Follow-up and endpoints

Clinical follow-up data were obtained from primary care records, hospital medical records, and postal questionnaires. Death certificates and autopsy reports were obtained. Follow-up duration was measured from the CMR date and truncated at 10 years. All events were adjudicated by a panel of experienced cardiologists who were blinded to CMR data. Patients were censored at the time of the first event. The primary endpoint was a composite of CV death or non-fatal major HF events [cardiac transplantation, left ventricular assist device (LVAD) implantation, or HF hospitalization]. Secondary endpoints were (i) all major HF events (composite of HF death and non-fatal HF events), (ii) CV death, and (iii) a sudden cardiac death (SCD) composite endpoint [(SCD or aborted SCD (aSCD)] (see Supplementary data online for full endpoint definitions).

### Statistical analysis

Patient characteristics are presented as frequencies (%) for categorical variables and median [interquartile range (IQR)] for continuous variables. Mann–Whitney test was used to compare continuous variables. Chi-squared test or Fisher’s exact test was used to compare categorical variables. Correlation between LA metrics was assessed using Pearson’s correlation coefficient. Linearity between LA parameters and the endpoints was assessed using restricted cubic splines with three knots placed at the 10th, 50th, and 90th percentiles. As all LA structure and function parameters were linearly associated with the endpoints (see Supplementary data online, *Figure S1*), these were considered as continuous variables in Cox proportional hazard models. Multivariable models adjusted for variables selected *a priori* on the basis of established association with adverse CV outcomes in DCM and included age, sex, New York Heart Association (NYHA) class, myocardial fibrosis presence, and LVEF.^12^ A sensitivity analysis included mitral regurgitation severity in addition to these pre-specified variables, to ensure independence of association between parameters of LA structure/function from mitral insufficiency. A second sensitivity analysis was also conducted that integrated LV global longitudinal strain (GLS) into the existing multivariable model, to ensure this did not attenuate the association between LA parameters and the primary endpoint. A pre-specified subgroup analysis was conducted in patients with mild–moderate DCM (patients with LVEF ≥ 35%) using the same multivariable model. Improvement in model performance was assessed using Harrel’s *C*-statistic, likelihood ratio test (LKR), Akaike information criterion, and both continuous and categorical net reclassification indices (NRIs). Arbitrary risk cut-offs of <15%, 15–30%, and >30% for categorical NRI were selected based on the incidence of events in the cohort. Intra-class correlation coefficient was used to assess intra- and inter-observer reproducibility. A two-tailed *P*-value of <0.05 was considered significant. Statistical analyses were conducted on Rstudio (v4.2.2): *survival* and *survminer* packages were used for survival analysis; figures were generated using *ggplot* package; and NRI was calculated using *nricens* package.

## Results

### Cohort

The primary cohort comprised 580 patients with confirmed DCM in sinus rhythm, of whom most were men [352 patients (61%)] and Caucasian [479 patients (83%)]. The median age was 54 (IQR 44–64) years. Indication for CMR included characterization of LV dysfunction in 431 patients (74%), investigation of arrhythmia in 47 patients (10%), and cardiomyopathy family screening in 35 patients (6%). The remaining 67 patients (12%) underwent CMR for other indications. Significant IHD was excluded by invasive coronary angiogram in 393 patients (68%), computed tomography coronary angiography in 41 patients (7%), and a functional test (stress perfusion CMR, nuclear scan, or stress echocardiogram) in 67 patients (12%). The remaining 79 patients were considered to have a very low clinical probability of IHD and did not undergo investigation to formally exclude: 51% were aged ≤40 years, none had prior angina, and none required revascularization or experienced an acute coronary syndrome during follow-up.

At baseline, 83% of patients were treated with angiotensin-converting enzyme inhibitors or angiotensin II receptor blockers, 70% with beta-blockers, and 37% with mineralocorticoid receptor antagonists. Compared with patients with LAVI_min_ below the median, those with LAVI_min_ above the median were older and had higher NYHA class, and a higher proportion were treated with HF drug therapies and had hypertension or a history of prior AF. Left and right ventricular volumes were higher whilst LVEF and right ventricular ejection fraction (RVEF) were lower in patients with LAVI_min_ above the median compared with those with LAVI_min_ below the median (*Table 1*). Patients with AF at the time of CMR excluded from the primary analysis (*n* = 105) were older, and a higher proportion were male, had hypertension, and were treated with beta-blockers and loop diuretics compared with those in sinus rhythm. LVEF and RVEF were lower in those with AF compared with those in sinus rhythm (see Supplementary data online, *Table S1*).

**Table 1:** Patient and cardiovascular magnetic resonance characteristics for the study cohort classified by LA minimum volume index above and below median

	All patients (*N* = 580)	LAVi_min_ ≤ median (*n* = 290)	LAVi_min_ > median (*n* = 290)	*P*-value
**Demographics**				
Age	54 (44–64)	52 (41–61)	56 (47–67)	**<0**.**0001**
Male	352 (61%)	172 (59%)	181 (62%)	0.44
Caucasian	479 (83%)	251 (87%)	228 (79%)	**0**.**019**
**Past medical history**				
Hypertension	170 (29%)	72 (25%)	98 (34%)	**0**.**018**
Diabetes mellitus	74 (13%)	32 (11%)	42 (14%)	0.21
Atrial fibrillation	52 (9%)	18 (6%)	34 (12%)	**0**.**012**
Smoker	61 (11%)	39 (13%)	22 (8%)	**0**.**021**
Excess alcohol	90 (16%)	43 (15%)	47 (16%)	0.77
Chemotherapy	26 (4%)	11 (4%)	15 (5%)	0.38
Peripartum presentation	10 (2%)	6 (2%)	4 (1%)	0.54
Neuromuscular disease	3 (0.5%)	2 (1%)	1 (0.3%)	0.59
Family history of DCM	91 (16%)	68 (23%)	23 (8%)	**<0**.**0001**
Family history of SCD	93 (16%)	57 (20%)	36 (12%)	**0**.**023**
**NYHA class**				
I	268 (46%)	152 (52%)	116 (40%)	**0**.**019**
II	218 (38%)	93 (32%)	125 (43%)	
III	87 (15%)	43 (15%)	44 (15%)	
IV	7 (1%)	2 (1%)	5 (2%)	
**Medication**				
Beta-blocker	406 (70%)	180 (62%)	226 (78%)	**<0**.**0001**
ACEi/ARB	482 (83%)	225 (78%)	257 (89%)	**0**.**0005**
Mineralocorticoid receptor antagonist	212 (37%)	85 (29%)	127 (44%)	**0**.**0003**
Loop diuretic	258 (44%)	97 (33%)	161 (56%)	**<0**.**0001**
**CMR characteristics**				
**Left ventricle**				
LVEDVi, mL/m^2^	119 (102–142)	108 (97–127)	132 (111–162)	**<0**.**0001**
LVESVi, mL/m^2^	135 (102–189)	56 (47–74)	85 (62–115)	**<0**.**0001**
LVMi, g/m^2^	85 (72–105)	79 (66–95)	94 (78–111)	**<0**.**0001**
LVSVi, mL/m^2^	49 (41–57)	50 (44–57)	47 (37–57)	**0**.**049**
LVEF, %	42 (30–51)	48 (40–53)	34 (26–46)	**<0**.**0001**
**Right ventricle**				
RVEDVi, mL/m^2^	84 (69–99)	81 (67–95)	89 (72–104)	**<0**.**0001**
RVESVi, mL/m^2^	38 (27–50)	34 (25–43)	43 (29–60)	**<0**.**0001**
RVSVi, mL/m^2^	45 (37–53)	47 (39–53)	42 (33–52)	**0**.**0009**
RVEF, %	55 (46–63)	58 (52–65)	51 (40–61)	**<0**.**0001**
**Mitral regurgitation**				
None	320 (55%)	211 (36%)	109 (19%)	**<0.0001**
Mild	192 (33%)	69 (12%)	123 (21%)	
Moderate	55 (9%)	9 (2%)	46 (8%)	
Severe	13 (2%)	1 (0.2%)	12 (2%)	
**Left atrium**				
LAVImin, mL/m^2^	22 (15–35)	15 (12–18)	35 (27–50)	**<0**.**0001**
LAVI_max_, mL/m^2^	47 (38–60)	38 (31–45)	60 (51–72)	**<0**.**0001**
LAEF, %	53 (38–61)	61 (56–65)	38 (27–49)	**<0**.**0001**
LARS, %	27.1 (17.0–35.4)	35.0 (29.2–41.1)	17.6 (11.5–24.5)	**<0**.**0001**
LABS, %	13.1 (8.1–18.4)	17.3 (13.2–22.9)	8.5 (5.4–13.0)	**<0**.**0001**
LACS, %	11.3 (6.2–19.0)	17.2 (10.2–23.7)	7.6 (3.7–12.8)	**<0**.**0001**
**Late gadolinium enhancement**	218 (38%)	88 (30%)	130 (45%)	**0**.**0005**

Data were presented as median (IQR) or *n* (%). Bold values in the “*P*-value column” refer to *p* values <0.05.

ACEi, angiotensin-converting enzyme inhibitor; ARB, angiotensin II receptor blocker; DCM, dilated cardiomyopathy; LA, left atrial; LABS, left atrial booster strain; LACS, left atrial conduit strain; LARS, left atrial reservoir strain; LAEF, left atrial ejection fraction; LAVI_max_, left atrial maximum volume index; LAVImin, left atrial minimum volume index; LVEDVi, left ventricular end-diastolic volume index; LVEF, left ventricular ejection fraction; LVESVi, left ventricular end-systolic volume index; LVMi, left ventricular mass index; NYHA, New York Heart Association; RVEDVi, right ventricular end-diastolic volume index; RVEF, right ventricular ejection fraction; RVESVi, right ventricular end-systolic volume index; SCD, sudden cardiac death.

### LA structure and function metrics

**Figure 1:**
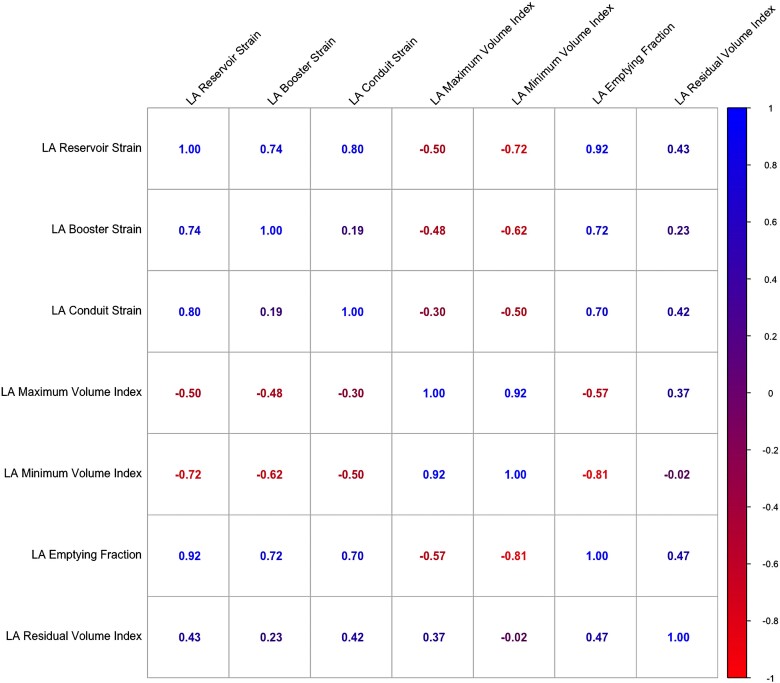
Correlation matrix for measures of LA structure and function derived from cardiovascular magnetic resonance in a cohort of patients with dilated cardiomyopathy in sinus rhythm. Correlation is depicted using colour scale for Pearson’s correlation coefficients. LA reservoir volume index was calculated as LA maximum volume minus LA minimum volume indexed to BSA. Variable levels of correlation were observed between measures of LA structure and function.

The level of correlation between measures of LA structure and function was variable, ranging from weak correlation between some parameters, including LACS with LABS (*r* = 0.19), to strong correlation between others, including LAVI_min_ with LAVI_max_ (*r* = 0.92) and LAEF with LARS (*r* = 0.92) (*Figure 1*). Intra-observer and inter-observer reproducibility for LA measures was good to excellent (see Supplementary data online, *Table S2*).

### Association between LA structure and function with CV mortality or major HF events

**Figure 2:**
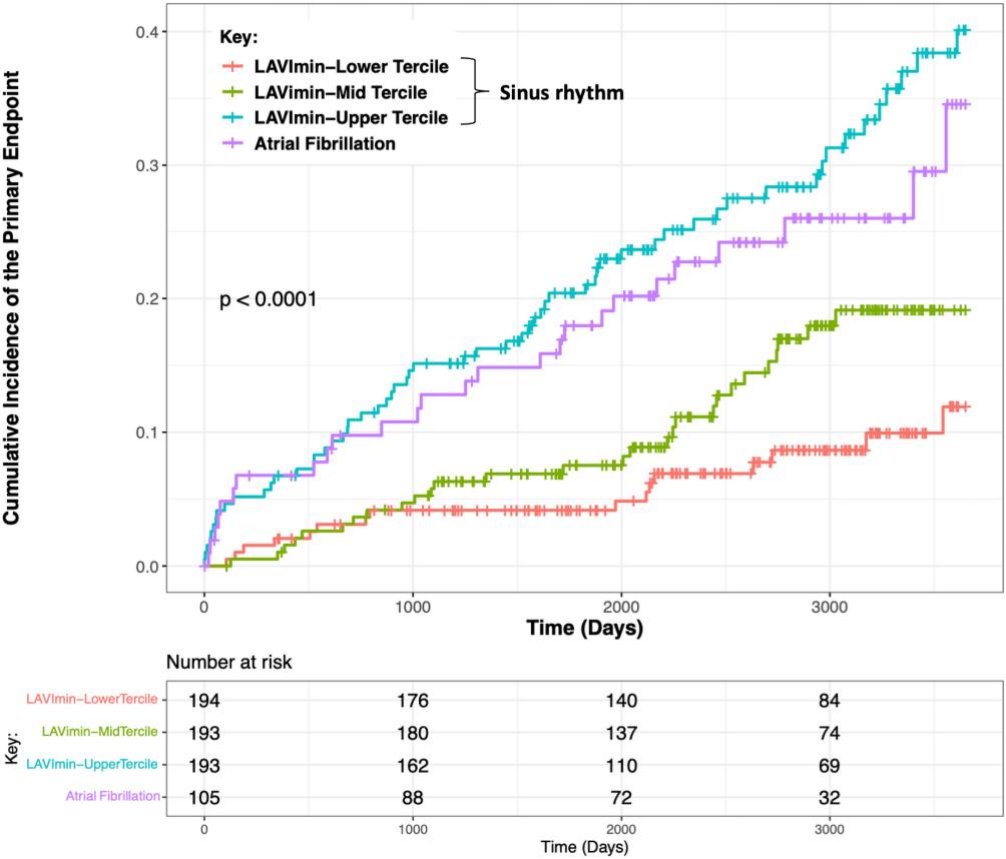
Cumulative incidence of the primary endpoint stratified by tercile of LA minimum volume index for patients with dilated cardiomyopathy in sinus rhythm; patients in AF at the time of cardiovascular magnetic resonance are included as a comparator group. Patients with LA minimum volume index in the highest tercile had higher cumulative incidence of the primary endpoint compared with middle and lowest terciles but a similar cumulative incidence for the primary endpoint compared with patients in AF.

Over a median follow-up of 7.4 years (IQR 4.7–9.3 years), 103 patients (18%) met the primary endpoint, including 20 patients (3.4%) who died from CV causes (8 from HF, 8 from SCD, and 4 from other CV causes) and 83 patients (14.3%) who had non-fatal HF events. On univariable analysis, all measures of LA structure and function were associated with the primary endpoint (*Table 2* and see Supplementary data online, *Table S3*). Each of these, apart from LACS, remained associated with the primary endpoint on multivariable analysis, adjusting for age, sex, NYHA class, fibrosis presence, and LVEF (*Table 2*). The addition of each LA metric to the multivariable model resulted in improved discrimination for the primary endpoint (*Table 3*), with the addition of LAVI_min_ offering the highest level of discrimination (*C*-statistic 0.702–0.738; *χ*^2^ test comparing LKR: *P* < 0.001). Thus, LAVI_min_ was taken forward as the LA parameter of choice for prognostic purposes. None of the measures of atrial strain improved model discrimination above those of LA volumes. The 5-year categorical NRI following the addition of LAVI_min_ to the baseline model was 0.210 (95% CI: 0.023–0.392), meaning an additional 17% of patients who had an event were reclassified to a more appropriate (higher) risk category and an additional 4% of patients who did not have an event were reclassified to a lower risk category (see Supplementary data online, *Table S4*). Patients in sinus rhythm with LAVI_min_ in the highest tercile had higher cumulative incidence of the primary endpoint compared with middle and lowest terciles (log-rank *P* < 0.0001) (*Figure 2*). Interestingly, patients in sinus rhythm with LAVI_min_ in the highest tercile had a similar cumulative incidence for the primary endpoint to patients in AF (*Figure 2*). In our first sensitivity analysis that additionally adjusted for mitral regurgitation, all LA parameters remained associated with the primary endpoint, with the addition of LAVI_min_ producing a similar improvement in model discrimination (*C*-statistic: 0.714–0.738; *χ*^2^ test comparing LKR: *P* < 0.001) (see Supplementary data online, *Tables S5* and *S6*). In our second sensitivity analysis that additionally adjusted for LV GLS, all LA parameters except LACS remained associated with the primary endpoint, again with the addition of LAVI_min_ resulting in similar improvement in model discrimination (*C*-statistic: 0.706–0.740; *χ*^2^ test comparing LKR: *P* < 0.001) (see Supplementary data online, *Tables S7* and *S8*).

**Table 2:** Univariable and multivariable associations between measures of LA structure and function with cardiovascular death or non-fatal major heart failure events

Characteristic	Univariable analysis		Multivariable qnalyses^a^	
	HR (95% CI)	*P*	HR (95% CI)	*P*
**LARS, per 10 units**	0.56 (0.47–0.67)	**<0**.**001**	0.66 (0.53–0.84)	**<0**.**001**
**LABS, per 10 units**	0.45 (0.33–0.61)	**<0**.**001**	0.58 (0.41–0.82)	**0**.**002**
**LACS, per 10 units**	0.50 (0.38–0.66)	**<0**.**001**	0.70 (0.49–1.00)	0.050
**LAEF, per 10%**	0.66 (0.59–0.74)	**<0**.**001**	0.74 (0.63–0.86)	**<0**.**001**
**LAVI_max_, per 10 mL/m^2^**	1.27 (1.18–1.36)	**<0**.**001**	1.21 (1.12–1.31)	**<0**.**001**
**LAVI_min_, per 10 mL/m^2^**	1.34 (1.25–1.44)	**<0**.**001**	1.27 (1.16–1.38)	**<0**.**001**

Cox proportional hazard models assessing the univariable and multivariable association between measures of LA structure and function with cardiovascular death or non-fatal major heart failure events. Bold values correspond to *p* values <0.05.

CI, confidence interval; HR, hazard ratio; LABS, left atrial booster strain; LACS, left atrial conduit strain; LARS, left atrial reservoir strain; LAEF, left atrial emptying fraction; LAVI_max_, left atrial maximum volume index; LAVImin, left atrial minimum volume index; LVEF, left ventricular ejection fraction; NYHA, New York Heart Association.

^a^Adjusted for age, sex, NYHA class, LVEF, and myocardial fibrosis presence

**Table 3:** Comparison of multivariable model discrimination for the primary endpoint integrating measures of left atrial structure and function

	*C*-statistic (95% CI)	AIC	*P*-value (*χ*^2^ test comparing LKR against Model 1)
**Model 1 (age + sex + NYHA class + LGE + LVEF)**	0.702 (0.651–0.753)	1195	−
**Model 2 (Model 1 + LARS)**	0.729 (0.680–0.778)	1184	**<0**.**001**
**Model 3 (Model 1 + LACS)**	0.710 (0.659–0.761)	1193	**0**.**046**
**Model 4 (Model 1 + LABS)**	0.724 (0.677–0.771)	1186	**0**.**001**
**Model 5 (Model 1 + LAEF)**	0.734 (0.685–0.783)	1182	**<0**.**001**
**Model 6 (Model 1 + LAVI_max_)**	0.732 (0.681–0.783)	1178	**<0**.**001**
**Model 7 (Model 1 + LAVI_min_)**	0.738 (0.687–0.789)	1174	**<0**.**001**

Bold values correspond to *p* values <0.05.

AIC, Akaike information criterion; CI, confidence interval; LABS, left atrial booster strain; LACS, left atrial conduit strain; LARS, left atrial reservoir strain; LAEF, left atrial emptying fraction; LAVI_max_, left atrial maximum volume index; LAVI_min_, left atrial minimum volume index; LGE, late gadolinium enhancement; LKR, likelihood ratio; LVEF, left ventricular ejection fraction; NYHA, New York Heart Association.

### Secondary endpoints

#### Major HF events

In total, 91 (16%) patients experienced a major HF event during follow-up. On univariable analysis, all measures of LA structure and function were associated with major HF events (see Supplementary data online, *Table S9*). Apart from LACS, all LA parameters remained associated with major HF events on multivariable analysis (see Supplementary data online, *Table S9*). As with the primary endpoint analysis, the degree of model improvement was similar between LA metrics, with LAVI_min_ offering the best discrimination (see Supplementary data online, *Table S10*).

#### CV death

In total, 48 (8%) patients died of CV causes during follow-up. As previous, all LA parameters were associated with CV mortality on univariable analysis. Each LA parameter, except LACS and LABS, remained associated with CV death on multivariable analysis (see Supplementary data online, *Table S11*). Model discrimination was similar for the LA parameters that remained associated with CV death on multivariable analysis, with LAVI_min_ offering the best discrimination (see Supplementary data online, *Table S12*).

#### Sudden cardiac death or aborted sudden cardiac death

In total, 38 (7%) patients met the composite SCD endpoint, including 8 SCDs and 30 aSCDs. All LA parameters, except LACS, were associated with this composite endpoint on univariable analysis (Supplementary data online, *Table S13A*). On multivariate analysis, these remained associated with the composite SCD endpoint. In contrast to earlier analyses, the addition of LABS resulted in the marginally greater improvement in model discrimination than the other LA parameters, including LAVI_min_ (see Supplementary data online, *Table S14*). Notably, guideline-based parameters used to determine primary prevention ICD implantation (LVEF < 35% and NYHA Class >I) were not associated with SCD/aSCD on univariable analysis (see Supplementary data online, *Table S13b*)

### The prognostic role of atrial structure and function in mild–moderate DCM

In the subgroup with mild–moderate DCM (*n* = 389), 50 (13%) met the primary endpoint (17 due to CV death and 33 due to non-fatal HF events). On univariate analysis, all LA parameters were associated with the primary endpoint. All but LACS remained associated on multivariable analysis (see Supplementary data online, *Table S15*). A similar level of discrimination was seen from the addition of each LA parameter on multivariable analysis, including LAVI_min_. In keeping with our other analyses, LA strain was not a superior discriminant for the primary endpoint to LA volumes in this subgroup (see Supplementary data online, *Table S16*).

## Discussion

### LA structure and function are independently associated with CV death and HF events in patients with DCM

In line with previous studies, we corroborate an important association between the left atrium and incident CV death or HF events in patients with DCM, independently of mitral regurgitation, LV GLS, and conventional markers of DCM phenotype severity. We take forward prior research in this field, including previous work from our group, through direct comparison of multiple measures of LA structure and function to determine parameters that best discriminate adverse CV events. This comparative approach distinguishes this paper from other recent studies evaluating LA parameters in patients with DCM.^10^ Whilst the added prognostic value was similar between the measures of LA structure and function in this cohort, LAVI_min_ was the best discriminator for the primary endpoint and additionally identified a subgroup of patients in sinus rhythm with a similar risk of CV death and major HF events to those in AF. These findings are important as LAVI_min_ is not currently a routine component of CMR reporting protocols. However, it is quick and reproducible to measure and would be simple to integrate into routine practice. It has been proposed that the added value of LAVI_min_ may relate to a degree of surrogacy for diastolic function, as it is measured at the point of direct continuum with LV end-diastolic pressure through the open mitral valve, and thus may be the most sensitive measure of LV filling pressure.^5^ LAVI_min_ also captures information on both LA size and contractile function, akin to LV end-systolic volume, which is a stronger prognostic indicator in HF than LV end-diastolic volume or LVEF.^13^ Supporting this hypothesis, we observed stronger correlation between LAVI_min_ than LAVI_max_ with all measures of LA function (*Figure 1*). Whether the small improvement in discrimination seen with LAVI_min_ compared with LAVI_max_ can enhance risk prediction and clinical outcomes requires further clarification.

### Clinical significance of study findings

The clinical application of our findings may include enhanced clinical decision-making through the identification of patients most likely to benefit from early consideration of add-on therapies for HF, including cardiac resynchronization, Vericiguat, or consideration for LVAD or transplantation. Additionally, these may help to identify patients with mild disease at higher risk of adverse CV outcomes, for whom introduction or intensification of drug therapies may be beneficial. A further important finding from this study was that LA strain did not improve risk prediction above LA volumetric analysis in this cohort. The additional analysis time and software required for LA strain measurement are therefore difficult to justify currently in this population.^10^

### LA parameters are associated with SCD in DCM

A major challenge in the clinical management of patients with DCM relates to risk prediction for SCD.^14^ As observed from other recent data,^15,16^ we found no association between LVEF or NYHA class and the SCD composite endpoint in this cohort. By contrast, we found that all measures of LA structure and function, apart from LACS, were associated with the SCD composite endpoint on multivariable analysis. The LA metric that enhanced the prediction of SCD/aSCD the most was LABS, which corroborates the work from Negishi *et al*.,^17^ who found LA booster pump function measured by echocardiography was independently associated with ventricular arrhythmias in patients with DCM who had an ICD. Collectively, these findings raise the question of whether LA parameters should be considered for arrhythmic risk stratification in DCM. Further work is required to evaluate utility in this capacity and study the mechanistic link between these indices and arrhythmogenesis. This finding parallels observations in HCM, where increased LA dimension is an established risk factor for SCD and used in clinical practice as a component of arrhythmic risk scoring tools.^18,19^

### Limitations

Patients in this study were enrolled from a single UK referral centre and its hospital network and the study inclusion criteria required a clinical referral for CMR, introducing a potential referral bias. The cohort was predominantly Caucasian patients (83%). Patients with AF at the time of CMR were excluded from the primary analysis as unreliable LA strain measurements in such cases were likely to confound results. A further limitation relates to the fact that CMR images to evaluate LA fibrosis were not obtained; thus, whether such sequences offer additional prognostic utility in DCM is unknown. However, there remains uncertainty about LA fibrosis reproducibility and thus clinical utility. A further limitation relates to the small level of incremental improvement in multivariable model performance from the addition of LAVI_min_ compared with LAVI_max_; larger multicentre studies are required to understand whether this small difference is clinically meaningful such that it improves prognostication and clinical outcomes. Finally, cardiac biomarkers, including natriuretic peptides, were not routinely measured, and it is possible that their inclusion in multivariable models may interact with the association between LA parameters and outcomes.

## Conclusion

LA structure and function are independently associated with adverse outcomes in patients with DCM. Volumetric analysis of the LA adequately captures risk and improves discrimination for CV death or major HF events; LA strain parameters do not offer incremental predictive value beyond these. LAVI_min_ may offer the greatest precision for risk prediction, but further validation is required. The role of LABS in the risk stratification of SCD requires further investigation.

## Supplementary Material

jeae080_Supplementary_Data

## Data Availability

The data are available from the corresponding author, on reasonable request. Data will be shared after review and approval by our Biobank scientific board, and terms of collaboration will be reached together with a signed data access agreement.
