# Supplementary material for: Comparative prognostic importance of measures of left atrial structure and function in non-ischaemic dilated cardiomyopathy
Source: Eur Heart J Cardiovasc Imaging. 2024 Mar 16;25(11):1566–74. doi: 10.1093/ehjci/jeae080 (PMC11522868; doi:10.1093/ehjci/jeae080)
Supplement: jeae080_Supplementary_Data [file jeae080_supplementary_data.docx]

**Hammersley DJ et al. Comparative prognostic importance of measures of left atrial structure and function in non-ischaemic dilated cardiomyopathy - Supplementary Data**

[Supplementary Methods 2](#_Toc136909513)

[Supplementary Tables 6](#_Toc136909514)

[Supplementary Figure 23](#_Toc136909515)

[Supplementary Material References 24](#_Toc136909516)

# Supplementary Methods

*Clinical endpoint definitions*

The following definitions were used by adjudicators for the purpose of adjudicating clinical endpoints in this study.^1,2^

*CV Death: Sudden Cardiac Death*

Death that occurs unexpectedly and not within 30 d of an acute MI. Sudden cardiac death includes the following scenarios:

1. Death witnessed and occurring without new or worsening symptoms.
2. Death witnessed within 60 min of the onset of new or worsening cardiac symptoms unless the symptoms suggest acute MI.
3. Death witnessed and attributed to an identified arrhythmia (e.g., captured on an electrocardiographic recording, witnessed on a monitor, or unwitnessed but found on ICD review).
4. Death after unsuccessful resuscitation from cardiac arrest (e.g., ICD unresponsive sudden cardiac death, pulseless electrical activity arrest).
5. Death after successful resuscitation from cardiac arrest and without identification of a specific cardiac or noncardiac aetiology.
6. Unwitnessed death in a subject seen alive and clinically stable ≤24 h before being found dead without any evidence supporting a specific non-cardiovascular cause of death (information about the patient’s clinical status preceding death should be provided if available)

Unless additional information suggests an alternate specific cause of death (e.g., Death due to Other Cardiovascular Causes), if a patient is seen alive ≤24 h before being found dead, sudden cardiac death (criterion [f]) should be recorded.

*CV Death: Acute MI*

Death by any cardiovascular mechanism (arrhythmia, sudden death, HF, stroke, pulmonary embolus, PAD) within 30 d after an acute MI, related to the immediate consequences of the

MI, such as progressive HF or recalcitrant arrhythmia. There may be assessable (attributable) mechanisms of cardiovascular death during this time period, but for simplicity, if the cardiovascular death occurs within 30 d of an acute MI, it will be considered a death due to MI.

Note: Acute MI should be verified to the extent possible by the diagnostic criteria outlined for acute MI or by autopsy findings showing recent MI or recent coronary thrombosis. Death resulting from a procedure to treat an MI (PCI or CABG), or to treat a complication resulting from MI, should also be considered death due to acute MI. Death resulting from an elective coronary procedure to treat myocardial ischemia (i.e., chronic stable angina) or death due to an MI that occurs as a direct consequence of a cardiovascular investigation / procedure / operation should be considered as a death due to a cardiovascular procedure.

*CV Death: HF*

Death associated with clinically worsening symptoms and/or signs of HF, regardless of HF aetiology.

*CV Death: CVA*

Death after a stroke that is either a direct consequence of the stroke or complication of stroke.

*CV Death: CV Procedure*

Death caused by the immediate complication(s) of a Cardiovascular procedure

*CV Death: CV Haemorrhage*

Death related to haemorrhage such as a non-stroke intracranial haemorrhage, (e.g., subdural hematoma) nonprocedural or nontraumatic vascular rupture (e.g., aortic aneurysm), or haemorrhage causing cardiac tamponade

*CV Death: Other*

Cardiovascular death not included in the above categories but with specific, known cause (e.g., pulmonary embolism).

*HF Hospitalisation*

An event where the patient is admitted to the hospital where each of the following criteria apply:

1. Primary diagnosis of HF.
2. Length of stay is at least 24 h (or extends over a calendar date if the hospital admission and discharge times are unavailable).
3. The patient exhibits new or worsening symptoms of HF on presentation and objective evidence of new or worsening HF.
4. Receives initiation or intensification of treatment specifically for HF.

*Aborted SCD*

Aborted SCD diagnosed if patients have received an appropriate implantable cardioverter-defibrillator (ICD) shock for ventricular arrhythmia, or had a nonfatal episode of ventricular fibrillation or spontaneous sustained ventricular tachycardia causing hemodynamic compromise and requiring cardioversion.

# Supplementary Tables

**Table S1: Patient and cardiovascular magnetic resonance characteristics of patients with atrial fibrillation at the time of cardiovascular magnetic resonance compared to patients in sinus rhythm**

|  | **Patients not in AF (N=580)** | **Patients in AF**  **(N=105)** | **P-value** |
| --- | --- | --- | --- |
| ***Demographics*** |  |  |  |
| Age | 54 (44-64) | 61 (52-68) | **<0.0001** |
| Male | 352 (61%) | 90 (86%) | **<0.0001** |
| Caucasian | 479 (83%) | 99 (94%) | **0.0024** |
| ***Past Medical History*** |  |  |  |
| Hypertension | 170 (29%) | 44 (42%) | **0.01** |
| Diabetes Mellitus | 74 (13%) | 10 (10%) | 0.35 |
| Smoker | 61 (11%) | 9 (9%) | 0.53 |
| Excess alcohol | 90 (16%) | 23 (22%) | 0.14 |
| Chemotherapy | 26 (4%) | 4 (4%) | 0.99 |
| Peripartum presentation | 10 (2%) | 0 (0%) | 0.38 |
| Neuromuscular disease | 3 (0.5%) | 0 (0%) | 0.99 |
| Family history of DCM | 91 (16%) | 7 (7%) | **0.0088** |
| Family history of SCD | 93 (16%) | 8 (8%) | **0.02** |
| ***NYHA Class*** |  |  |  |
| I | 268 (46%) | 33 (31%) | 0.068 |
| II | 218 (38%) | 54 (52%) |  |
| III | 87 (15%) | 18 (17%) |  |
| IV | 7 (1%) | 0 (0%) |  |
| ***Medication*** |  |  |  |
| Beta-Blocker | 406 (70%) | 87 (83%) | **0.0046** |
| ACEi/ARNI | 482 (83%) | 91 (87%) | 0.28 |
| Mineralocorticoid receptor antagonist | 212 (37%) | 45 (43%) | 0.21 |
| Loop diuretic | 258 (44%) | 66 (63%) | **0.0006** |
| ***CMR Characteristics*** |  |  |  |
| ***Left Ventricle*** |  |  |  |
| LVEDVi, ml/m^2^ | 119 (102.3-142.0) | 114 (101.3-138) | 0.19 |
| LVESVi, ml/m^2^ | 135 (102-189) | 80.5 (61-95.8) | **0.02** |
| LVMi, g/m^2^ | 85 (71.50-105) | 86 (75-100) | 0.63 |
| LVSVi, ml/m^2^ | 49 (41-57) | 36 (29-45) | **<0.0001** |
| LVEF, % | 42 (30-51) | 31 (23-39) | **<0.0001** |
| ***Right Ventricle*** |  |  |  |
| RVEDVi, ml/m^2^ | 84 (69-99) | 84.5 (68.5-101.8) | 0.82 |
| RVESVi, ml/m^2^ | 38 (27-50) | 47.5 (36.3-61) | **<0.0001** |
| RVSVi, ml/m^2^ | 45 (37-53) | 36 (29-41.8) | **<0.0001** |
| RVEF, % | 55 (46-63) | 41.5 (33.3-50) | **<0.0001** |
| ***Mitral Regurgitation*** |  |  | **0.0003** |
| None | 320 (55%) | 39 (37%) |  |
| Mild | 192 (33%) | 41 (39%) |  |
| Moderate | 55 (9%) | 23 (22%) |  |
| Severe | 13 (2%) | 2 (2%) |  |
| ***Late gadolinium enhancement*** | 218 (38%) | 38 (36%) | 0.79 |
| Data presented as median (IQR) or n (%). ACEi = angiotensin-converting enzyme inhibitor; ARB = angiotensin II receptor blocker; DCM = dilated cardiomyopathy; LA = left atrial; LABS = left atrial booster strain; LACS = left atrial conduit strain; LARS = left atrial reservoir strain; LAEF = left atrial emptying fraction; LAVImax = left atrial maximum volume index; LAVImin = left atrial minimum volume index; LVEDVi = left ventricular end-diastolic volume index; LVEF = left ventricular ejection fraction; LVESVi = left ventricular end-systolic volume index; LVMi = left ventricular mass index; NYHA = New York Heart Association; RVEDVi = right ventricular end-diastolic volume index; RVEF = right ventricular ejection fraction; RVESVi = right ventricular end-systolic volume index; SCD = sudden cardiac death; VT = ventricular tachycardia. | | | |

**Table S2: Intra-observer and inter-observer variability for measures of left atrial structure and function**

|  | **Intra-observer variability** | | | **Inter-observer variability** | | |
| --- | --- | --- | --- | --- | --- | --- |
|  | **ICC** | **95% CI** | **p** | **ICC** | **95% CI** | **p** |
| LAVImax | 0.987 | 0.971-0.994 | **<0.0001** | 0.969 | 0.936-0.985 | **<0.0001** |
| LAVImin | 0.990 | 0.986-0.997 | **<0.0001** | 0.974 | 0.943-0.988 | **<0.0001** |
| LAEF | 0.975 | 0.938-0.989 | **<0.0001** | 0.889 | 0.778-0.946 | **<0.0001** |
| LARS | 0.947 | 0.892-0.974 | **<0.0001** | 0.885 | 0.758-0.945 | **<0.0001** |
| LACS | 0.912 | 0.826-0.957 | **<0.0001** | 0.839 | 0.689-0.920 | **<0.0001** |
| LABS | 0.923 | 0.846-0.963 | **<0.0001** | 0.876 | 0.806-0.973 | **<0.0001** |
| CI = confidence interval; ICC = intraclass correlation coefficient; LABS = left atrial booster strain; LACS = left atrial conduit strain; LARS = left atrial reservoir strain; LAEF = left atrial emptying fraction; LAVImax = left atrial maximum volume index; LAVImin = left atrial minimum volume index | | | | | | |

**Table S3: Univariable and multivariable associations with cardiovascular death or non-fatal major heart failure events**

| **Univariable** | | | **Multivariable Models** | | | | | | | | | | | | | |
| --- | --- | --- | --- | --- | --- | --- | --- | --- | --- | --- | --- | --- | --- | --- | --- | --- |
|  | | | **Model 1** | | **Model 2** | | **Model 3** | | **Model 4** | | **Model 5** | | **Model 6** | | **Model 7** | |
| **Characteristic** | **HR (95%CI)** | **P** | **HR (95%CI)** | **P** | **HR (95%CI)** | **P** | **HR (95%CI)** | **P** | **HR (95%CI)** | **P** | **HR (95%CI)** | **P** | **HR (95%CI)** | **P** | **HR (95%CI)** | **P** |
| **Age, per 10 years** | 1.06 (0.92–1.22) | 0.392 | 1.02 (0.88–1.18) | 0.781 | 1.01 (0.87–1.17) | 0.916 | 1.08 (0.93–1.25) | 0.310 | 0.97 (0.83–1.13) | 0.709 | 1.03 (0.89–1.19) | 0.686 | 1.03 (0.90–1.19) | 0.656 | 1.05 (0.91–1.21) | 0.523 |
| **Male** | 1.19 (0.79–1.78) | 0.405 | 1.11 (0.72–1.70) | 0.634 | 1.09 (0.71–1.68) | 0.681 | 1.09 (0.71–1.68) | 0.689 | 1.12 (0.73–1.72) | 0.606 | 1.09 (0.71–1.67) | 0.699 | 1.11 (0.72–1.71) | 0.624 | 1.12 (0.73–1.72) | 0.613 |
| **NYHA II** | 1.65 (1.04 –2.63) | **0.034** | 1.32 (0.81–2.14) | 0.263 | 1.31 (0.81–2.13) | 0.269 | 1.31 (0.81–2.12) | 0.277 | 1.33 (0.82–2.16) | 0.250 | 1.27 (0.78–2.06) | 0.341 | 1.36 (0.84–2.20) | 0.215 | 1.30 (0.80–2.12) | 0.287 |
| **NYHA Class III/IV** | 3.29 (2.10–5.40) | **<0.001** | 2.39 (1.41–4.05) | **0.001** | 2.33 (1.37–3.96) | **0.002** | 2.41 (1.42–4.10) | **0.001** | 2.36 (1.39–4.01) | **0.001** | 2.30 (1.35–3.90) | **0.002** | 2.57 (1.51–4.37) | **<0.001** | 2.41 (1.42–4.10) | **0.001** |
| **LVEF,** **per 10%** | 0.62 (0.53–0.73) | **<0.001** | 0.69 (0.58–0.81) | **<0.001** | 0.88 (0.71–1.08) | 0.230 | 0.79 (0.66–0.94) | **0.009** | 0.78 (0.64–0.95) | **0.015** | 0.89 (0.72–1.09) | 0.251 | 0.76 (0.64–0.89) | **0.001** | 0.83 (0.69–0.99) | **0.034** |
| **LGE** | 1.90 (1.29–2.79) | **0.001** | 1.62 (1.09–2.42) | **0.017** | 1.59 (1.07–2.36) | **0.023** | 1.57 (1.05–2.33) | **0.027** | 1.65 (1.11–2.45) | **0.014** | 1.62 (1.09–2.42) | **0.017** | 1.51 (1.01–2.25) | **0.043** | 1.55 (1.04–2.31) | **0.030** |
| **LARS, per 10 units** | 0.56 (0.47–0.67) | **<0.001** |  |  | 0.66 (0.53–0.84) | **<0.001** |  |  |  |  |  |  |  |  |  |  |
| **LABS, per 10 units** | 0.45 (0.33–0.61) | **<0.001** |  |  |  |  | 0.58 (0.41–0.82) | **0.002** |  |  |  |  |  |  |  |  |
| **LACS, per 10 units** | 0.50 (0.38–0.66) | **<0.001** |  |  |  |  |  |  | 0.70 (0.49–1.00) | 0.050 |  |  |  |  |  |  |
| **LAEF, per 10%** | 0.66 (0.59–0.74) | **<0.001** |  |  |  |  |  |  |  |  | 0.74 (0.63–0.86) | **<0.001** |  |  |  |  |
| **LAVImax, per 10ml/m^2^** | 1.27 (1.18–1.36) | **<0.001** |  |  |  |  |  |  |  |  |  |  | 1.21 (1.12–1.31) | **<0.001** |  |  |
| **LAVImin, per 10ml/m^2^** | 1.34 (1.25–1.44) | **<0.001** |  |  |  |  |  |  |  |  |  |  |  |  | 1.27 (1.16–1.38) | **<0.001** |
| HR = hazard ratio; LABS = left atrial booster strain; LACS = left atrial conduit strain; LARS = left atrial reservoir strain; LAEF = left atrial emptying fraction; LAVImax = left atrial maximum volume index; LAVImin = left atrial minimum volume index; LGE = late gadolinium enhancement; LVEF = left ventricular ejection fraction | | | | | | | | | | | | | | | | |

**Table S4: Categorical and continuous net reclassification table assessing the incremental predictive value from the addition of left atrial minimum volume index to a multivariable model in relation to the primary endpoint of cardiovascular death or major heart failure events**

|  |  | **LVEF + NYHA + LGE + Age + Sex + LAVImin** | | | |  |  |
| --- | --- | --- | --- | --- | --- | --- | --- |
| **5-year risk** | | **0-15%** | **15-30%** | **30%+** | **Total** | **Higher** | **Lower** |
| **LVEF + NYHA + LGE + Age +Sex** | |  |  |  |  |  |  |
| **CV mortality/ Major HF events** | |  |  |  |  |  |  |
|  | **0-15%** | 20 | 7 | 1 | 28 | 8 | NA |
|  | **15-30%** | 8 | 9 | 10 | 27 | 10 | 8 |
|  | **30%+** | 0 | 0 | 5 | 5 | NA | 0 |
|  | **Total** | 28 | 16 | 16 | 60 | 18 | 8 |
| **No CV mortality/ Major HF events** | |  |  |  |  |  |  |
|  | **0-15%** | 306 | 11 | 2 | 319 | 13 | NA |
|  | **15-30%** | 37 | 35 | 12 | 84 | 12 | 37 |
|  | **30%+** | 0 | 3 | 6 | 9 | NA | 3 |
|  | **Total** | 343 | 49 | 20 | 412 | 25 | 40 |
|  |  |  |  |  |  |  |  |
|  |  | **NRI** | **95% CI (bootstrap method)** |  |  |  |  |
|  | **Categorical NRI** |  |  |  |  |  |  |
|  | Event NRI | 0.170 | 0.007-0.357 |  |  |  |  |
|  | Non-event NRI | 0.040 | -0.010-0.082 |  |  |  |  |
|  | Overall NRI | 0.210 | 0.023-0.392 |  |  |  |  |
|  | **Continuous NRI** |  |  |  |  |  |  |
|  | Event NRI | 0.008 | -0.232-0.252 |  |  |  |  |
|  | Non-event NRI | 0.407 | 0.211-0.547 |  |  |  |  |
|  | Overall NRI | 0.415 | 0.057-0.718 |  |  |  |  |

CI = confidence interval; CV = cardiovascular; HF = heart failure; LAVImin = left atrial minimum volume index; LGE = late gadolinium enhancement; LVEF = left ventricular ejection fraction; NRI = net reclassification index; NYHA = New York Heart Association.

**Table S5: Sensitivity analysis integrating mitral regurgitation severity into multivariable models evaluating the association between each measure of left atrial structure and function with the primary endpoint of cardiovascular death or major heart failure events**

| **Multivariable Models** | | | | | | | | | | | | | |  |
| --- | --- | --- | --- | --- | --- | --- | --- | --- | --- | --- | --- | --- | --- | --- |
| **Characteristic** | **Model 1** | | **Model 2** | | **Model 3** | | **Model 4** | | **Model 5** | | **Model 6** | | **Model 7** | |
|  | **HR (95%CI)** | **P** | **HR (95%CI)** | **P** | **HR (95%CI)** | **P** | **HR (95%CI)** | **P** | **HR (95%CI)** | **P** | **HR (95%CI)** | **P** | **HR (95%CI)** | **P** |
| **Age, per 10 years** | 1.02 (0.88 – 1.18) | 0.764 | 1.01 (0.87 – 1.17) | 0.887 | 1.08 (0.93 – 1.25) | 0.336 | 0.98 (0.84 – 1.14) | 0.799 | 1.03 (0.89 – 1.19) | 0.683 | 1.03 (0.89 – 1.19) | 0.671 | 1.05 (0.91 – 1.21) | 0.534 |
| **Male** | 1.18 (0.76 – 1.82) | 0.465 | 1.13 (0.73 – 1.75) | 0.584 | 1.14 (0.73 – 1.76) | 0.562 | 1.17 (0.76 – 1.81) | 0.476 | 1.11 (0.72 – 1.72) | 0.628 | 1.12 (0.72 – 1.74) | 0.617 | 1.12 (0.72 – 1.73) | 0.623 |
| **NYHA II** | 1.30 (0.80 – 2.11) | 0.294 | 1.30 (0.80 – 2.12) | 0.281 | 1.30 (0.80 – 2.11) | 0.291 | 1.31 (0.80 – 2.12) | 0.280 | 1.26 (0.78 – 2.05) | 0.347 | 1.35 (0.83 – 2.20) | 0.222 | 1.30 (0.80 – 2.12) | 0.290 |
| **NYHA Class III/IV** | 2.40 (1.40 – 4.10) | **0.001** | 2.33 (1.36 – 3.98) | **0.002** | 2.43 (1.42 – 4.15) | **0.001** | 2.35 (1.38 – 4.01) | **0.002** | 2.29 (1.34 – 3.92) | **0.003** | 2.58 (1.51 – 4.42) | **0.001** | 2.42 (1.42 – 4.13) | **0.001** |
| **LVEF,** **per 10%** | 0.74 (0.61 – 0.89) | **0.001** | 0.89 (0.72 – 1.11) | 0.293 | 0.81 (0.67 – 0.98) | **0.034** | 0.81 (0.66 – 1.00) | 0.055 | 0.89 (0.72 – 1.11) | 0.301 | 0.77 (0.64 – 0.92) | **0.005** | 0.83 (0.68 – 1.00) | 0.053 |
| **LGE** | 1.58 (1.06 – 2.35) | **0.024** | 1.57 (1.05 – 2.33) | **0.027** | 1.54 (1.04 – 2.30) | **0.033** | 1.61 (1.08 – 2.39) | **0.020** | 1.61 (1.08 – 2.40) | **0.019** | 1.50 (1.01 – 2.24) | **0.045** | 1.55 (1.04 – 2.31) | **0.031** |
| **Mild MR** | 1.34 (0.84 – 2.16) | 0.221 | 1.10 (0.68 – 1.80) | 0.691 | 1.18 (0.73 – 1.91) | 0.501 | 1.26 (0.78 – 2.04) | 0.335 | 1.05 (0.63 – 1.73) | 0.859 | 1.11 (0.68 – 1.79) | 0.680 | 1.03 (0.63 – 1.68) | 0.910 |
| **Moderate MR** | 1.63 (0.85 – 3.10) | 0.138 | 1.27 (0.65 – 2.46) | 0.481 | 1.37 (0.71 – 2.64) | 0.346 | 1.49 (0.78 – 2.87) | 0.226 | 1.19 (0.61 – 2.32) | 0.611 | 1.08 (0.54 – 2.14) | 0.836 | 1.01 (0.51 – 2.01) | 0.982 |
| **Severe MR** | 2.06 (0.79 – 5.37) | 0.137 | 1.43 (0.53 – 3.81) | 0.479 | 1.53 (0.58 – 4.06) | 0.390 | 1.86 (0.71 – 4.88) | 0.207 | 1.31 (0.49 – 3.53) | 0.592 | 1.04 (0.36 – 3.01) | 0.946 | 0.99 (0.34 – 2.83) | 0.978 |
| **LARS, per 10 units** |  |  | 0.68 (0.54 – 0.87) | **0.002** |  |  |  |  |  |  |  |  |  |  |
| **LABS, per 10 units** |  |  |  |  | 0.61 (0.43 – 0.87) | **0.006** |  |  |  |  |  |  |  |  |
| **LACS, per 10  units** |  |  |  |  |  |  | 0.74 (0.52 – 1.06) | 0.098 |  |  |  |  |  |  |
| **LAEF, per 10%** |  |  |  |  |  |  |  |  | 0.75 (0.63 – 0.88) | **0.001** |  |  |  |  |
| **LAVImax, per 10ml/m^2^** |  |  |  |  |  |  |  |  |  |  | 1.21 (1.10 – 1.33) | **<0.001** |  |  |
| **LAVImin, per 10ml/m^2^** |  |  |  |  |  |  |  |  |  |  |  |  | 1.27 (1.15 – 1.40) | **<0.001** |
| HR = hazard ratio; LABS = left atrial booster strain; LACS = left atrial conduit strain; LARS = left atrial reservoir strain; LAEF = left atrial emptying fraction; LAVImax = left atrial maximum volume index; LAVImin = left atrial minimum volume index; LGE = late gadolinium enhancement; LVEF = left ventricular ejection fraction, MR = Mitral Regurgitation | | | | | | | | | | | | | | |

**Table S6: Comparison of multivariable model discrimination for the primary endpoint integrating mitral regurgitation severity into multivariable models including each measure of left atrial structure and function**

|  | **C-statistic (95% CI)** | **AIC** | **P-value (χ2 test comparing LKR against Model 1)** |
| --- | --- | --- | --- |
| **Model 1 (Age + Sex+ NYHA class + LGE + LVEF + mitral regurgitation severity)** | 0.714  (0.665-0.763) | 1197 | - |
| **Model 2 (Model 1 + LARS)** | 0.731  (0.682-0.780) | 1189 | **<0.001** |
| **Model 3 (Model 1 + LACS)** | 0.719  (0.670-0.768) | 1197 | 0.093 |
| **Model 4 (Model 1 + LABS)** | 0.729  (0.682-0.776) | 1191 | **0.004** |
| **Model 5 (Model 1 + LAEF)** | 0.735  (0.686-0.784) | 1187 | **<0.001** |
| **Model 6 (Model 1 + LAVImax)** | 0.733  (0.682-0.784) | 1184 | **<0.001** |
| **Model 7 (Model 1 + LAVImin)** | 0.738  (0.687-0.789) | 1181 | **<0.001** |
| CI = confidence interval; LABS = left atrial booster strain; LACS = left atrial conduit strain; LARS = left atrial reservoir strain; LAEF = left atrial emptying fraction; LAVImax = left atrial maximum volume index; LAVImin = left atrial minimum volume index; LGE = late gadolinium enhancement; AIC = Akaike Information Criterion; LKR = Likelihood Ratio; LVEF = left ventricular ejection fraction; NYHA = New York Heart Association. | | | |

**Table S7: Sensitivity analysis integrating left ventricular global longitudinal strain into multivariable models evaluating the association between each measure of left atrial structure and function with the primary endpoint of cardiovascular death or major heart failure events**

| **Characteristic** | **Multivariable Analyses*** | |
| --- | --- | --- |
|  | **HR (95%CI)** | **P** |
| **LARS, per 10 units** | **0.67 (0.53–0.86)** | **0.002** |
| **LABS, per 10 units** | **0.60 (0.42–0.85)** | **0.004** |
| **LACS, per 10 units** | **0.75 (0.52–1.09)** | 0.130 |
| **LAEF, per 10%** | **0.74 (0.63–0.87)** | **<0.001** |
| **LAVImax, per 10ml/m^2^** | **1.21 (1.11–1.31)** | **<0.001** |
| **LAVImin, per 10ml/m^2^** | **1.26 (1.15–1.38)** | **<0.001** |
| Cox proportional hazard models assessing multivariable association between measures of left atrial structure and function with cardiovascular death or non-fatal major heart failure events.  *Adjusted for Age, Sex, NYHA class, LV GLS, LVEF and myocardial fibrosis presence  CI = confidence interval; HR = Hazard ratio; LABS = left atrial booster strain; LACS = left atrial conduit strain; LARS = left atrial reservoir strain; LAEF = left atrial emptying fraction; LAVImax = left atrial maximum volume index; LAVImin = left atrial minimum volume index; LV GLS = left ventricular global longitudinal strain; LVEF = Left Ventricular Ejection Fraction; NYHA = New York Heart Association. | | |

**Table S8: Comparison of multivariable model discrimination for the primary endpoint integrating LV GLS into multivariable models including each measure of left atrial structure and function**

|  | **C-statistic (95% CI)** | **AIC** | **P-value (χ2 test comparing LKR against Model 1)** |
| --- | --- | --- | --- |
| **Model 1 (Age + Sex+ NYHA class + LGE + LV GLS + LVEF)** | 0.706  (0.657-0.755) | 1194 | - |
| **Model 2 (Model 1 + LARS)** | 0.730  (0.681-0.779) | 1186 | **0.001** |
| **Model 3 (Model 1 + LACS)** | 0.713  (0.664-0.762) | 1193 | 0.124 |
| **Model 4 (Model 1 + LABS)** | 0.727  (0.678-0.774) | 1187 | **0.003** |
| **Model 5 (Model 1 + LAEF)** | 0.734  (0.685-0.783) | 1183 | **<0.001** |
| **Model 6 (Model 1 + LAVImax)** | 0.737  (0.688-0.786) | 1179 | **<0.001** |
| **Model 7 (Model 1 + LAVImin)** | 0.740  (0.691-0.789) | 1176 | **<0.001** |
| AIC = Akaike Information Criterion; CI = confidence interval; LABS = left atrial booster strain; LACS = left atrial conduit strain; LARS = left atrial reservoir strain; LAEF = left atrial emptying fraction; LAVImax = left atrial maximum volume index; LAVImin = left atrial minimum volume index; LGE = late gadolinium enhancement; LKR = Likelihood Ratio; LV GLS = left ventricular global longitudinal strain; LVEF = Left Ventricular Ejection Fraction; NYHA = New York Heart Association. | | | |

**Table S9: Univariable and multivariable associations between each measure of left atrial structure and function with heart failure events**

| **Univariable** | | | **Multivariable Models** | | | | | | | | | | | | | |
| --- | --- | --- | --- | --- | --- | --- | --- | --- | --- | --- | --- | --- | --- | --- | --- | --- |
|  | | | **Model 1** | | **Model 2** | | **Model 3** | | **Model 4** | | **Model 5** | | **Model 6** | | **Model 7** | |
| **Characteristic** | **HR (95%CI)** | **P** | **HR (95%CI)** | **P** | **HR (95%CI)** | **P** | **HR (95%CI)** | **P** | **HR (95%CI)** | **P** | **HR (95%CI)** | **P** | **HR (95%CI)** | **P** | **HR (95%CI)** | **P** |
| **Age, per 10 years** | 1.06 (0.92 – 1.24) | 0.411 | 1.02 (0.87 – 1.19) | 0.826 | 1.01 (0.86 – 1.18) | 0.909 | 1.08 (0.92 – 1.27) | 0.337 | 0.97 (0.82 – 1.14) | 0.705 | 1.03 (0.88 – 1.20) | 0.685 | 1.03 (0.89 – 1.20) | 0.687 | 1.05 (0.90 – 1.22) | 0.543 |
| **Male** | 1.02 (0.67 – 1.56) | 0.932 | 0.97 (0.62 – 1.52) | 0.890 | 0.96 (0.61 – 1.50) | 0.851 | 0.96 (0.61 – 1.50) | 0.845 | 0.98 (0.63 – 1.53) | 0.922 | 0.95 (0.61 – 1.49) | 0.827 | 0.97 (0.62 – 1.53) | 0.907 | 0.98 (0.62 – 1.54) | 0.927 |
| **NYHA II** | 1.90 (1.14 –3.17) | **0.014** | 1.46 (0.86 – 2.48) | 0.164 | 1.45 (0.85 – 2.46) | 0.169 | 1.44 (0.85 – 2.46) | 0.175 | 1.47 (0.86 – 2.50) | 0.155 | 1.39 (0.82 – 2.36) | 0.225 | 1.49 (0.88 – 2.54) | 0.141 | 1.42 (0.84 – 2.43) | 0.194 |
| **NYHA Class III/IV** | 4.06 (2.39 – 6.90) | **<0.001** | 2.77 (1.57 – 4.88) | **<0.001** | 2.69 (1.52 – 4.74) | **0.001** | 2.79 (1.58 – 4.94) | **<0.001** | 2.73 (1.55 – 4.81) | **0.001** | 2.64 (1.49 – 4.66) | **0.001** | 2.98 (1.69 – 5.27) | **<0.001** | 2.79 (1.58 – 4.92) | **<0.001** |
| **LVEF,** **per 10%** | 0.60 (0.51– 0.71) | **<0.001** | 0.67 (0.56 – 0.79) | **<0.001** | 0.86 (0.69 – 1.07) | 0.181 | 0.76 (0.63 – 0.93) | **0.007** | 0.76 (0.61 – 0.94) | **0.011** | 0.87 (0.70 – 1.09) | 0.221 | 0.73 (0.61 – 0.88) | **0.001** | 0.80 (0.66 – 0.97) | **0.022** |
| **LGE** | 1.83 (1.21 – 2.76) | **0.004** | 1.59 (1.04 – 2.43) | **0.031** | 1.56 (1.02 – 2.39) | **0.038** | 1.54 (1.01 – 2.36) | **0.044** | 1.62 (1.06 – 2.47) | **0.026** | 1.61 (1.05 – 2.46) | **0.028** | 1.48 (0.97 – 2.25) | 0.072 | 1.52 (1.00 – 2.33) | 0.051 |
| **LARS, per 10 units** | 0.54 (0.44 – 0.65) | **<0.001** |  |  | 0.65 (0.51 – 0.84) | **0.001** |  |  |  |  |  |  |  |  |  |  |
| **LABS, per 10 units** | 0.42 (0.30 – 0.59) | **<0.001** |  |  |  |  | 0.57 (0.39 – 0.82) | **0.003** |  |  |  |  |  |  |  |  |
| **LACS, per 10  units** | 0.47 (0.35 – 0.64) | **<0.001** |  |  |  |  |  |  | 0.70 (0.48 – 1.02) | 0.062 |  |  |  |  |  |  |
| **LAEF, per 10%** | 0.64 (0.57 – 0.72) | **<0.001** |  |  |  |  |  |  |  |  | 0.72 (0.61 – 0.85) | **<0.001** |  |  |  |  |
| **LAVImax, per 10ml/m^2^** | 1.27 (1.18 – 1.37) | **<0.001** |  |  |  |  |  |  |  |  |  |  | 1.22 (1.12 – 1.32) | **<0.001** |  |  |
| **LAVImin, per 10ml/m^2^** | 1.35 (1.26– 1.45) | **<0.001** |  |  |  |  |  |  |  |  |  |  |  |  | 1.27 (1.16 – 1.39) | **<0.001** |
| HR = hazard ratio; LABS = left atrial booster strain; LACS = left atrial conduit strain; LARS = left atrial reservoir strain; LAEF = left atrial emptying fraction; LAVImax = left atrial maximum volume index; LAVImin = left atrial minimum volume index; LGE = late gadolinium enhancement; LVEF = left ventricular ejection fraction | | | | | | | | | | | | | | | | |

**Table S10: Comparison of multivariable model discrimination for major heart failure events integrating measures of left atrial structure and function**

|  | **C-statistic (95% CI)** | **AIC** | **P-value (χ2 test comparing LKR against Model 1)** |
| --- | --- | --- | --- |
| **Model 1 (Age + Sex+ NYHA class + LGE + LVEF)** | 0.718  (0.665-0.771) | 1052 | - |
| **Model 2 (Model 1 + LARS)** | 0.747  (0.698-0.796) | 1042 | **<0.001** |
| **Model 3 (Model 1 + LACS)** | 0.726  (0.675-0.777) | 1051 | 0.056 |
| **Model 4 (Model 1 + LABS)** | 0.742  (0.693-0.791) | 1044 | **0.002** |
| **Model 5 (Model 1 + LAEF)** | 0.752  (0.703-0.801) | 1039 | **<0.001** |
| **Model 6 (Model 1 + LAVImax)** | 0.753  (0.702-0.804) | 1037 | **<0.001** |
| **Model 7 (Model 1 + LAVImin)** | 0.758  (0.707-0.809) | 1033 | **<0.001** |
| CI = confidence interval; LABS = left atrial booster strain; LACS = left atrial conduit strain; LARS = left atrial reservoir strain; LAEF = left atrial emptying fraction; LAVImax = left atrial maximum volume index; LAVImin = left atrial minimum volume index; LGE = late gadolinium enhancement; AIC = Akaike Information Criterion; LKR = Likelihood Ratio; LVEF = left ventricular ejection fraction; NYHA = New York Heart Association. | | | |

**Table S11: Univariable and multivariable associations between measures of left atrial structure and function with cardiovascular death**

| **Univariable** | | | **Multivariable Models** | | | | | | | | | | | | | |  |
| --- | --- | --- | --- | --- | --- | --- | --- | --- | --- | --- | --- | --- | --- | --- | --- | --- | --- |
|  | | | **Model 1** | | **Model 2** | | **Model 3** | | **Model 4** | | **Model 5** | | **Model 6** | | **Model 7** | |  |
| **Characteristic** | **HR (95%CI)** | **P** | **HR (95%CI)** | **P** | **HR (95%CI)** | **P** | **HR (95%CI)** | **P** | **HR (95%CI)** | **P** | **HR (95%CI)** | **P** | **HR (95%CI)** | **P** | **HR (95%CI)** | **P** |  |
| **Age, per 10 years** | 1.31 (1.05 –1.63) | 0.015 | 1.29 (1.03 – 1.62) | **0.025** | 1.27 (1.01 – 1.59) | **0.038** | 1.34 (1.07 – 1.68) | **0.012** | 1.23 (0.97 – 1.55) | 0.088 | 1.29 (1.03 – 1.61) | **0.026** | 1.25 (1.01 – 1.56) | **0.045** | 1.26 (1.01 – 1.57) | **0.038** |  |
| **Male** | 1.29 (0.71 –2.36) | 0.401 | 1.19 (0.63 – 2.24) | 0.590 | 1.19 (0.63 – 2.24) | 0.596 | 1.19 (0.63 – 2.25) | 0.589 | 1.20 (0.63 – 2.25) | 0.580 | 1.20 (0.64 – 2.26) | 0.576 | 1.35 (0.70 – 2.58) | 0.369 | 1.35 (0.70 – 2.58) | 0.370 |  |
| **NYHA II** | 1.49 (0.74 – 2.98) | 0.261 | 1.08 (0.53 – 2.22) | 0.832 | 1.08 (0.53 – 2.22) | 0.829 | 1.09 (0.53 – 2.23) | 0.815 | 1.08 (0.53 – 2.21) | 0.839 | 1.06 (0.52 – 2.18) | 0.871 | 1.15 (0.56 – 2.37) | 0.700 | 1.11 (0.54 – 2.28) | 0.781 |  |
| **NYHA Class III/IV** | 3.56 (1.76 – 7.22) | **<0.001** | 2.61 (1.23 – 5.53) | **0.013** | 2.67 (1.26 – 5.68) | **0.011** | 2.69 (1.26 – 5.73) | **0.010** | 2.62 (1.24 – 5.55) | **0.012** | 2.65 (1.25 – 5.66) | **0.011** | 2.92 (1.36 – 6.29) | **0.006** | 2.78 (1.30 – 5.98) | **0.009** |  |
| **LVEF,** **per 10%** | 0.65 (0.52 – 0.82) | **<0.001** | 0.73 (0.58 – 0.93) | **0.010** | 0.90 (0.67 – 1.21) | 0.482 | 0.81 (0.62 – 1.05) | 0.108 | 0.83 (0.63 – 1.10) | 0.195 | 0.89 (0.67 – 1.20) | 0.453 | 0.80 (0.63 – 1.02) | 0.073 | 0.86 (0.67 – 1.10) | 0.226 |  |
| **LGE** | 2.70 (1.51 – 4.81) | **<0.001** | 2.26 (1.25 – 4.11) | **0.007** | 2.21 (1.22 – 4.00) | **0.009** | 2.16 (1.19 – 3.92) | **0.011** | 2.32 (1.28 – 4.22) | **0.006** | 2.24 (1.23 – 4.05) | **0.008** | 2.08 (1.15 – 3.77) | **0.015** | 2.14 (1.18 – 3.88) | **0.012** |  |
| **LARS, per 10 units** | 0.58 (0.44 – 0.75) | **<0.001** |  |  | 0.69 (0.49 – 0.96) | **0.029** |  |  |  |  |  |  |  |  |  |  |  |
| **LABS, per 10 units** | 0.54 (0.35 – 0.84) | **0.006** |  |  |  |  | 0.65 (0.40 – 1.06) | 0.085 |  |  |  |  |  |  |  |  |  |
| **LACS, per 10  units** | 0.46 (0.30 – 0.70) | **<0.001** |  |  |  |  |  |  | 0.67 (0.39 – 1.14) | 0.142 |  |  |  |  |  |  |  |
| **LAEF, per 10%** | 0.69 (0.58 – 0.82) | **<0.001** |  |  |  |  |  |  |  |  | 0.77 (0.62 – 0.97) | **0.023** |  |  |  |  |  |
| **LAVImax, per 10ml/m^2^** | 1.23 (1.14 – 1.34) | **<0.001** |  |  |  |  |  |  |  |  |  |  | 1.20 (1.08 – 1.33) | **0.001** |  |  |  |
| **LAVImin, per 10ml/m^2^** | 1.27 (1.17 – 1.38) | **<0.001** |  |  |  |  |  |  |  |  |  |  |  |  | 1.23 (1.10 – 1.37) | **<0.001** |  |
| HR = hazard ratio; LABS = left atrial booster strain; LACS = left atrial conduit strain; LARS = left atrial reservoir strain; LAEF = left atrial emptying fraction; LAVImax = left atrial maximum volume index; LAVImin = left atrial minimum volume index; LGE = late gadolinium enhancement; LVEF = left ventricular ejection fraction | | | | | | | | | | | | | | | | | |

**Table S12: Comparison of multivariable model discrimination for cardiovascular death integrating measures of left atrial structure and function**

|  | **C-statistic (95% CI)** | **AIC** | **P-value (χ2 test comparing LKR against Model 1)** |
| --- | --- | --- | --- |
| **Model 1 (Age + Sex+ NYHA class + LGE + LVEF)** | 0.744  (0.671- 0.817) | 547 | - |
| **Model 2 (Model 1 + LARS)** | 0.754  (0.680- 0.828) | 544 | **0.025** |
| **Model 3 (Model 1 + LACS)** | 0.748  (0.675- 0.821) | 546 | 0.132 |
| **Model 4 (Model 1 + LABS)** | 0.748  (0.674- 0.822) | 545 | 0.072 |
| **Model 5 (Model 1 + LAEF)** | 0.753  (0.679- 0.827) | 543 | **0.023** |
| **Model 6 (Model 1 + LAVImax)** | 0.769  (0.700- 0.838) | 540 | **0.003** |
| **Model 7 (Model 1 + LAVImin)** | 0.770  (0.701- 0.839) | 539 | **0.002** |
| CI = confidence interval; LABS = left atrial booster strain; LACS = left atrial conduit strain; LARS = left atrial reservoir strain; LAEF = left atrial emptying fraction; LAVImax = left atrial maximum volume index; LAVImin = left atrial minimum volume index; LGE = late gadolinium enhancement; AIC = Akaike Information Criterion; LKR = Likelihood Ratio; LVEF = left ventricular ejection fraction; NYHA = New York Heart Association. | | | |

**Table 13a: Univariable and multivariable associations between measures of left atrial structure and function with sudden cardiac death or aborted sudden cardiac death**

| **Univariable** | | | **Multivariable Models** | | | | | | | | | | | | | |
| --- | --- | --- | --- | --- | --- | --- | --- | --- | --- | --- | --- | --- | --- | --- | --- | --- |
|  | | | **Model 1** | | **Model 2** | | **Model 3** | | **Model 4** | | **Model 5** | | **Model 6** | | **Model 7** | |
| **Characteristic** | **HR (95%CI)** | **P** | **HR (95%CI)** | **P** | **HR (95%CI)** | **P** | **HR (95%CI)** | **P** | **HR (95%CI)** | **P** | **HR (95%CI)** | **P** | **HR (95%CI)** | **P** | **HR (95%CI)** | **P** |
| **Age, per 10 years** | 1.02 (0.81 –1.28) | 0.863 | 1.00 (0.79 – 1.27) | 0.989 | 0.95 (0.74 – 1.22) | 0.692 | 1.05 (0.83 – 1.34) | 0.677 | 0.96 (0.74 – 1.24) | 0.737 | 0.97 (0.77 – 1.24) | 0.818 | 0.98 (0.78 – 1.24) | 0.883 | 0.98 (0.78 – 1.24) | 0.870 |
| **Male** | 2.11 (1.00 – 4.45) | 0.051 | 1.46 (0.65 – 3.24) | 0.358 | 1.46 (0.65 – 3.25) | 0.356 | 1.44 (0.64 – 3.20) | 0.376 | 1.47 (0.66 – 3.27) | 0.345 | 1.45 (0.65 – 3.23) | 0.370 | 1.52 (0.68 – 3.41) | 0.308 | 1.54 (0.69 – 3.47) | 0.295 |
| **NYHA II** | 0.65 (0.29 –1.45) | 0.294 | 0.51 (0.21 – 1.22) | 0.131 | 0.51 (0.21 – 1.21) | 0.124 | 0.50 (0.21 – 1.20) | 0.122 | 0.51 (0.22 – 1.23) | 0.135 | 0.49 (0.20 – 1.16) | 0.106 | 0.53 (0.22 – 1.26) | 0.152 | 0.51 (0.21 – 1.22) | 0.130 |
| **NYHA Class III/IV** | 2.04 (0.96 –4.32) | 0.063 | 1.95 (0.87 – 4.39) | 0.106 | 2.03 (0.90 – 4.57) | 0.086 | 2.04 (0.91 – 4.59) | 0.084 | 1.99 (0.89 – 4.49) | 0.096 | 2.01 (0.89 – 4.54) | 0.092 | 2.13 (0.94 – 4.84) | 0.071 | 2.06 (0.91 – 4.66) | 0.084 |
| **LVEF,** **per 10%** | 0.84 (0.65 –1.08) | 0.180 | 0.95 (0.73 – 1.22) | 0.675 | 1.27 (0.90 – 1.79) | 0.169 | 1.17 (0.87 – 1.58) | 0.304 | 1.04 (0.76 – 1.44) | 0.797 | 1.30 (0.93 – 1.82) | 0.130 | 1.03 (0.79 – 1.35) | 0.819 | 1.13 (0.84 – 1.52) | 0.406 |
| **LGE** | 5.69 (2.68 –12.06) | **<0.001** | 5.32 (2.45 – 11.53) | **<0.001** | 5.12 (2.36 – 11.08) | **<0.001** | 5.16 (2.38 – 11.20) | **<0.001** | 5.31 (2.45 – 11.51) | **<0.001** | 5.32 (2.46 – 11.55) | **<0.001** | 5.10 (2.35 – 11.06) | **<0.001** | 5.20 (2.40 – 11.29) | **<0.001** |
| **LARS, per 10 units** | 0.67 (0.51 –0.89) | **0.005** |  |  | 0.61 (0.41 – 0.90) | **0.013** |  |  |  |  |  |  |  |  |  |  |
| **LABS, per 10 units** | 0.47 (0.28 –0.77) | **0.003** |  |  |  |  | 0.42 (0.22 – 0.80) | **0.008** |  |  |  |  |  |  |  |  |
| **LACS, per 10  units** | 0.75 (0.50 –1.12) | 0.160 |  |  |  |  |  |  | 0.76 (0.44 – 1.33) | 0.336 |  |  |  |  |  |  |
| **LAEF, per 10%** | 0.75 (0.62 –0.90) | **0.003** |  |  |  |  |  |  |  |  | 0.68 (0.52 – 0.88) | **0.004** |  |  |  |  |
| **LAVImax, per 10ml/m^2^** | 1.12 (1.04 –1.31) | **0.010** |  |  |  |  |  |  |  |  |  |  | 1.17 (1.00 – 1.35) | **0.046** |  |  |
| **LAVImin, per 10ml/m^2^** | 1.20 (1.07 –1.35) | **0.002** |  |  |  |  |  |  |  |  |  |  |  |  | 1.24 (1.06 – 1.46) | **0.009** |
| HR = hazard ratio; LABS = left atrial booster strain; LACS = left atrial conduit strain; LARS = left atrial reservoir strain; LAEF = left atrial emptying fraction; LAVImax = left atrial maximum volume index; LAVImin = left atrial minimum volume index; LGE = late gadolinium enhancement; LVEF = left ventricular ejection fraction | | | | | | | | | | | | | | | | |

**Table 13b: Univariable associations between current guideline-recommended arrhythmic risk parameters and sudden death or aborted sudden death events in this cohort**

| **Variable** | **HR (95% CI)** | **P-value** |
| --- | --- | --- |
| **NYHA Class > I** | 1.04 (0.55-1.97) | 0.902 |
| **LVEF <35%** | 1.30 (0.67-2.52] | 0.431 |
| CI = confidence interval; HR = hazard ratio; LVEF = left ventricular ejection fraction; NYHA = New York Heart Association | | |

**Table S14: Comparison of multivariable model discrimination for the sudden cardiac death composite endpoint integrating measures of left atrial structure and function**

|  | **C-statistic (95% CI)** | **AIC** | **P-value (χ2 test comparing LKR against Model 1)** |
| --- | --- | --- | --- |
| **Model 1 (Age + Sex+ NYHA class + LGE + LVEF)** | 0.772  (0.648-0.796) | 418 | - |
| **Model 2 (Model 1 + LARS)** | 0.789  (0.724- 0.854) | 413 | **0.011** |
| **Model 3 (Model 1 + LACS)** | 0.777  (0.706- 0.848) | 419 | 0.323 |
| **Model 4 (Model 1 + LABS)** | 0.791  (0.717- 0.865) | 412 | **0.005** |
| **Model 5 (Model 1 + LAEF)** | 0.789  (0.718- 0.860) | 412 | **0.005** |
| **Model 6 (Model 1 + LAVImax)** | 0.778  (0.705- 0.851) | 417 | 0.061 |
| **Model 7 (Model 1 + LAVImin)** | 0.785  (0.716- 0.854) | 415 | **0.017** |
| CI = confidence interval; LABS = left atrial booster strain; LACS = left atrial conduit strain; LARS = left atrial reservoir strain; LAEF = left atrial emptying fraction; LAVImax = left atrial maximum volume index; LAVImin = left atrial minimum volume index; LGE = late gadolinium enhancement; AIC = Akaike Information Criterion; LVEF = left ventricular ejection fraction; NYHA = New York Heart Association. | | | |

**Table S15: Univariable and multivariable associations between measures of left atrial structure and function with cardiovascular death or non-fatal heart failure events in the subgroup of patients with dilated cardiomyopathy and left ventricular ejection fraction ≥35%**

| **Univariable** | | | **Multivariable Models** | | | | | | | | | | | | | |
| --- | --- | --- | --- | --- | --- | --- | --- | --- | --- | --- | --- | --- | --- | --- | --- | --- |
|  | | | **Model 1** | | **Model 2** | | **Model 3** | | **Model 4** | | **Model 5** | | **Model 6** | | **Model 7** | |
| **Characteristic** | **HR (95%CI)** | **P** | **HR (95%CI)** | **P** | **HR (95%CI)** | **P** | **HR (95%CI)** | **P** | **HR (95%CI)** | **P** | **HR (95%CI)** | **P** | **HR (95%CI)** | **P** | **HR (95%CI)** | **P** |
| **Age, per 10 years** | 1.25 (1.02 – 1.54) | **0.035** | 1.19 (0.96 – 1.48) | 0.117 | 1.10 (0.88 – 1.36) | 0.407 | 1.25 (1.01 – 1.55) | **0.038** | 1.10 (0.87 – 1.40) | 0.429 | 1.13 (0.92 – 1.40) | 0.250 | 1.12 (0.90 – 1.39) | 0.311 | 1.12 (0.90 – 1.38) | 0.302 |
| **Male** | 1.08 (0.61 – 1.91) | 0.796 | 1.24 (0.68 – 2.27) | 0.491 | 1.27 (0.69 – 2.35) | 0.446 | 1.25 (0.68 – 2.30) | 0.472 | 1.26 (0.68 – 2.32) | 0.458 | 1.28 (0.69 – 2.36) | 0.434 | 1.36 (0.73 – 2.52) | 0.336 | 1.42 (0.76 – 2.66) | 0.275 |
| **NYHA II** | 1.39 (0.71 – 2.70) | 0.333 | 1.00 (0.49 – 2.02) | 0.992 | 0.99 (0.49 – 2.01) | 0.977 | 0.99 (0.49 – 2.00) | 0.968 | 1.01 (0.50 – 2.05) | 0.979 | 0.97 (0.48 – 1.97) | 0.936 | 1.01 (0.50 – 2.06) | 0.975 | 1.00 (0.49 – 2.04) | 0.999 |
| **NYHA Class III/IV** | 3.88 (1.97 –  7.63) | **<0.001** | 2.78 (1.31 – 5.92) | **0.008** | 2.66 (1.24 – 5.71) | **0.012** | 2.99 (1.38 – 6.46) | **0.005** | 2.70 (1.27 – 5.75) | **0.010** | 2.54 (1.17 – 5.52) | **0.018** | 3.16 (1.45 – 6.87) | **0.004** | 2.89 (1.33 – 6.28) | **0.007** |
| **LVEF,** **per 10%** | 0.46 (0.31 – 0.70) | **0.0003** | 0.53 (0.34 – 0.81) | **0.003** | 0.70 (0.44 – 1.11) | 0.131 | 0.62 (0.40 – 0.96) | **0.031** | 0.59 (0.38 – 0.93) | **0.022** | 0.69 (0.43 – 1.09) | 0.110 | 0.54 (0.35 – 0.83) | **0.005** | 0.60 (0.39 – 0.93) | **0.022** |
| **LGE** | 1.82 (1.05 – 3.18) | **0.034** | 1.36 (0.76 – 2.43) | 0.307 | 1.33 (0.75 – 2.36) | 0.330 | 1.24 (0.69 – 2.21) | 0.475 | 1.41 (0.79 – 2.52) | 0.249 | 1.36 (0.76 – 2.41) | 0.302 | 1.26 (0.71 – 2.25) | 0.436 | 1.32 (0.74 – 2.35) | 0.345 |
| **LARS, per 10 units** | 0.52 (0.39 – 0.67) | **<0.001** |  |  | 0.60 (0.45 – 0.81) | **0.001** |  |  |  |  |  |  |  |  |  |  |
| **LABS, per 10 units** | 0.43 (0.27 – 0.68) | **<0.001** |  |  |  |  | 0.43 (0.27 – 0.71) | **0.001** |  |  |  |  |  |  |  |  |
| **LACS, per 10  units** | 0.54 (0.37 – 0.79) | **0.001** |  |  |  |  |  |  | 0.73 (0.47 – 1.14) | 0.169 |  |  |  |  |  |  |
| **LAEF, per 10%** | 0.61 (0.51 – 0.73) | **<0.001** |  |  |  |  |  |  |  |  | 0.67 (0.55 – 0.82) | **<0.001** |  |  |  |  |
| **LAVImax, per 10ml/m^2^** | 1.23 (1.10 – 1.38) | **<0.001** |  |  |  |  |  |  |  |  |  |  | 1.23 (1.08 – 1.39) | **0.001** |  |  |
| **LAVImin, per 10ml/m^2^** | 1.37 (1.23 – 1.53) | **<0.001** |  |  |  |  |  |  |  |  |  |  |  |  | 1.33 (1.17 – 1.50) | **<0.001** |
| HR = hazard ratio; LABS = left atrial booster strain; LACS = left atrial conduit strain; LARS = left atrial reservoir strain; LAEF = left atrial emptying fraction; LAVImax = left atrial maximum volume index; LAVImin = left atrial minimum volume index; LGE = late gadolinium enhancement; LVEF = left ventricular ejection fraction | | | | | | | | | | | | | | | | |

**Table S16: Comparison of multivariable model discrimination for the primary endpoint integrating measures of left atrial structure and function for patients with dilated cardiomyopathy and left ventricular ejection fraction ≥35%**

|  | **C-statistic (95% CI)** | **AIC** | **P-value (χ2 test comparing LKR against Model 1)** |
| --- | --- | --- | --- |
| **Model 1 (Age + Sex+ NYHA class + LGE + LVEF)** | 0.696  (0.618- 0.774) | 550 | - |
| **Model 2 (Model 1 + LARS)** | 0.728  (0.654- 0.802) | 541 | **<0.001** |
| **Model 3 (Model 1 + LACS)** | 0.699  (0.623- 0.775) | 551 | 0.164 |
| **Model 4 (Model 1 + LABS)** | 0.727  (0.654- 0.800) | 540 | **<0.001** |
| **Model 5 (Model 1 + LAEF)** | 0.733  (0.657- 0.809) | 538 | **<0.001** |
| **Model 6 (Model 1 + LAVImax)** | 0.733  (0.655- 0.811) | 544 | **0.004** |
| **Model 7 (Model 1 + LAVImin)** | 0.736  (0.658- 0.814) | 538 | **<0.001** |
| CI = confidence interval; LABS = left atrial booster strain; LACS = left atrial conduit strain; LARS = left atrial reservoir strain; LAEF = left atrial emptying fraction; LAVImax = left atrial maximum volume index; LAVImin = left atrial minimum volume index; LGE = late gadolinium enhancement; AIC = Akaike Information Criterion; LVEF = left ventricular ejection fraction; NYHA = New York Heart Association. | | | |

# Supplementary Figure

**Figure S1: Restricted Cubic splines used to explore the linearity of association between measures of left atrial structure and function and the primary endpoint, adjusted for age, sex, NYHA class, myocardial fibrosis presence and LVEF.** P value for nonlinearity was ≥0.05 for each measure of left atrial structure and function

**
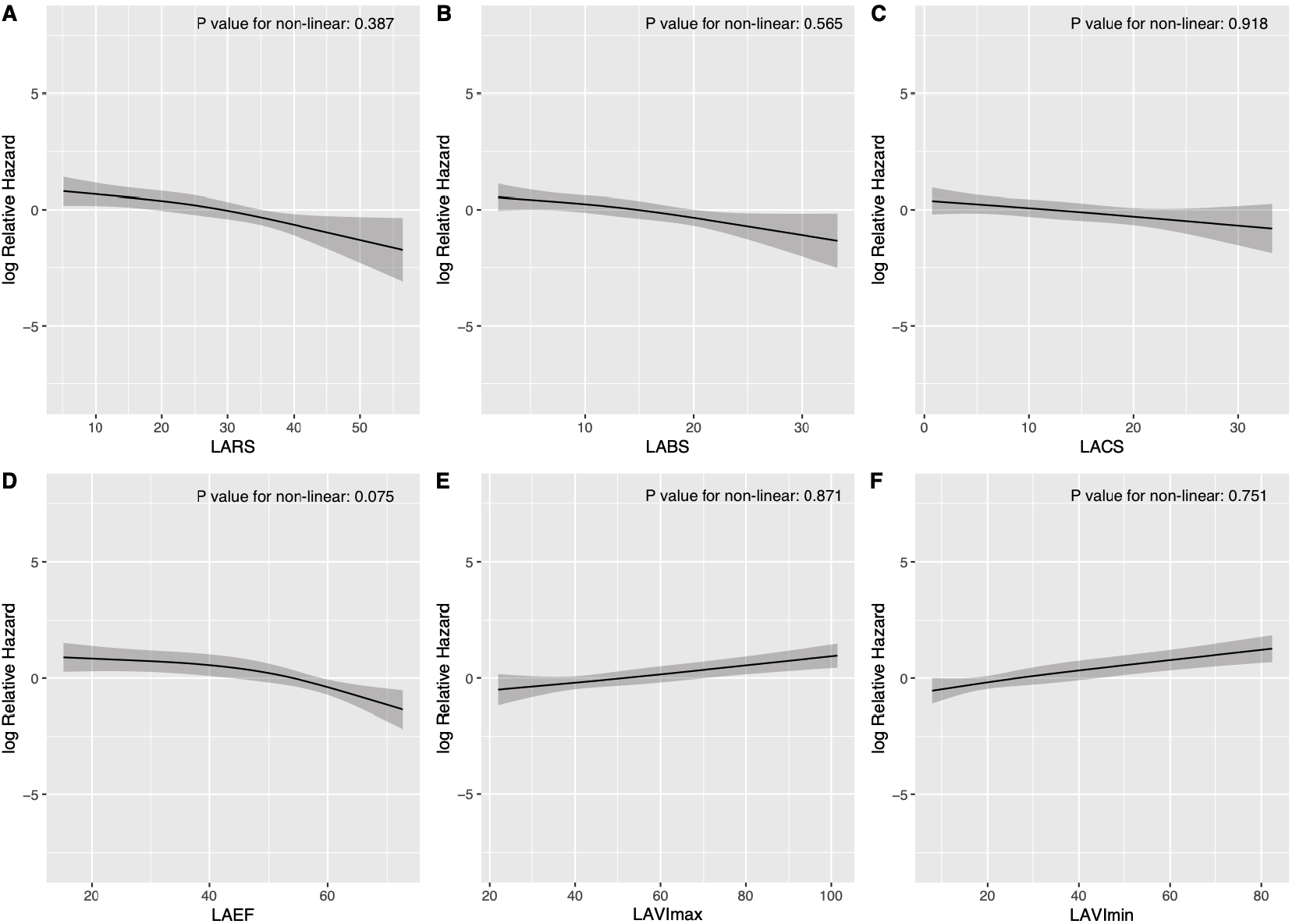
**

LARS = Left Atrial Reservoir strain; LACS = Left Atrial Conduit strain; LABS = Left Atrial Booster strain; LAEF = Left Atrial Emptying Fraction; LA.Max.vol.i = Left Atrial Maximum Volume index; LA.min.vol.i = Left Atrial Minimum Volume

# Supplementary Material References

1. Hicks KA, Tcheng JE, Bozkurt B, Chaitman BR, Cutlip DE, Farb A, Fonarow GC, Jacobs JP, Jaff MR, Lichtman JH, Limacher MC, Mahaffey KW, Mehran R, Nissen SE, Smith EE, Targum SL. 2014 ACC/AHA key data elements and definitions for cardiovascular endpoint events in clinical trials: A Report of the American College of Cardiology/American Heart Association Task Force on Clinical Data Standards (Writing Committee to Develop Cardiovascu. *J Am Coll Cardiol* 2015;**66**:403–469.

2. American College of Cardiology/American Heart Association Task Force on Clinical Data Standards (ACC/AHA/HRS Writing Committee to Develop Data Standards on Electrophysiology), Buxton AE, Calkins H, Callans DJ, DiMarco JP, Fisher JD, Greene HL, Haines DE, Hayes DL, Heidenreich PA, Miller JM, Poppas A, Prystowsky EN, Schoenfeld MH, Zimetbaum PJ, Goff DC, Grover FL, Malenka DJ, Peterson ED, Radford MJ, Redberg RF. ACC/AHA/HRS 2006 key data elements and definitions for electrophysiological studies and procedures: a report of the American College of Cardiology/American Heart Association Task Force on Clinical Data Standards (ACC/AHA/HRS Writing Committee to Develop D. *Circulation* 2006;**114**:2534–2570.
